# Benefits of Bilateral Bone Conduction Device Use Including Osia Devices in Children and Adolescents With Bilateral Atresia

**DOI:** 10.1177/23312165261422955

**Published:** 2026-02-18

**Authors:** Robel Z. Alemu, Alan Blakeman, Jaina Negandhi, Blake C. Papsin, Sharon L. Cushing, Karen A. Gordon

**Affiliations:** 1 Archie's Cochlear Implant Laboratory, 7979 The Hospital for Sick Children, Toronto, Canada; 2 Institute of Medical Science, The University of Toronto, Toronto, Canada; 3 Department of Otolaryngology-Head & Neck Surgery, University of Toronto, Toronto, Canada; 4 Department of Otolaryngology, 7979 The Hospital for Sick Children, Toronto, Canada; 5 Program in Neuroscience and Mental Health, Research Institute, Toronto, Canada; 6 Department of Communication Disorders, 7979 The Hospital for Sick Children, Toronto, Canada

**Keywords:** bone conduction hearing, Osia, Baha Connect, transcutaneous, percutaneous, bilateral conductive hearing loss and atresia, sound localization/spatial hearing, unrestricted head movements, speech perception and spatial release from masking, SSQ

## Abstract

This study aimed to characterize effects of bilateral bone conduction devices (BCD) including the Cochlear™ Osia^®^ (Osia) and the Cochlear™ percutaneous Baha^®^ Connect System (Baha) on localization of stationary and moving sound in children and adolescents with bilateral atresia. Participants were 11 listeners with BCDs [*M*_Age_(SD) = 14.7(3.5) years] and 11 age-matched controls [*M*_Age_(SD) = 14.9(1.9) years]. Outcomes were word recognition in quiet and noise, spatial release from masking (SRM) [spondee-word recognition thresholds in noise at co-located/0° or separated (90° left/right) positions], self-reported hearing using the Speech, Spatial and Qualities of Hearing Scale (SSQ), and localization of stationary and moving sound with tracking of real-time unrestricted head movements. BCD users had reduced speech perception accuracy in noise during unilateral listening (*p* < .001) and higher speech recognition thresholds than controls (*p* = .001). BCD users had higher errors than controls during stationary (*p* < .001) and moving (*p* < .001) sound localization consistent with self-reported spatial hearing challenges. BCD users had significantly reduced errors during bilateral use compared to unilateral use for stationary (*p* < .01) but not always for moving (right unilateral: *p* < .01; left unilateral: *p* = .46) sound localization. BCD users spent less time moving their heads in the correct direction compared to controls for stationary and moving sound localization (*p* < .01). Results indicate that children and adolescents with BCDs demonstrate improved localization of stationary but not moving sound-sources, with bilateral device use compared to unilateral use. This finding provides evidence for some access to binaural cues and mitigation of head shadow despite transcranial attenuation, but ineffective use of head movements.

## Introduction

Bone conduction devices (BCDs) are a common treatment for children with atresia (absence of the external ear canal) who are unable to use traditional hearing aids. Congenital aplasia or hypoplasia of the external auditory canal (Fuchsmann et al., 2010), occurs in approximately two in every 10,000 births and is commonly associated with microtia (abnormal or absent pinna) (Shaw et al., 2004). These malformations can be unilateral or bilateral, have a higher incidence in boys than girls, and are associated with both syndromic and nonsyndromic genetic mutations (Billings et al., 2016) or vascular insults and teratogens (Kelley & Scholes, 2007). In children, early treatment using BCDs, of the accompanying conductive hearing loss is needed to promote development of speech and language (Fuchsmann et al., 2010).

BCDs provide hearing through vibrations of the skull (osseous temporal bone) in response to auditory stimulation which is translated by bone conduction to the ipsilateral and contralateral cochleae (Stevens et al., 2025). The difference in bone conduction transmission sensitivity between the two sides (transcranial attenuation) can be variable between individuals (Stenfelt, 2012). There are five mechanisms of bone conduction hearing including: (1) sound radiation to the external ear canal, (2) middle ear ossicle inertia, (3) inertia of the cochlear fluids, (4) compression of the cochlear walls, and (5) pressure transmission from the cerebrospinal fluid, with inertia of cochlear fluids considered the most important mechanism (Stenfelt & Goode, 2005) as the inner ear creates traveling waves on the basilar membrane.

The present study focused on children and adolescents using bilateral surgical BCDs. Notably, there are nonsurgical BCDs which are placed on the head through external attachment mechanism such as a headband, softband, adhesive, or eyeglasses; however, the nonsurgical devices suffer from signal attenuation at high frequencies as well as limitations to prolonged wear time and retention (Ellsperman et al., 2021; Verstraeten et al., 2009). Surgical conduction devices, either percutaneous or transcutaneous, can alleviate some of these issues.

Percutaneous BCDs are one type of surgical BCD. A processor converting sound to vibration is fit to a percutaneous abutment that is osseointegrated with the skull (Robinette et al., 2023). Drawbacks include esthetic concerns, need for daily hygiene care, soft tissue overgrowth of the abutment, infection, skin reaction, and loss of the implant (Chan et al., 2017; Kiringoda & Lustig, 2013). In children, the age of implant is also an important consideration particularly due to immature skull structure and thin bone thickness (Davids et al., 2007; Mierzwiński et al., 2015). Transcutaneous devices, designed to resolve these problems, use an implant which is completely contained under the skin (Ellsperman et al., 2021; Key et al., 2024). Passive transcutaneous devices have an external component which magnetically interfaces with and drives vibration of the implant (Cushing et al., 2024; Ellsperman et al., 2021; Polonenko et al., 2016). Passive transcutaneous BCDs suffer from skin and soft tissue impedance with signal attenuation of up to 20 dB at high frequencies (Briggs et al., 2015; Siegert & Kanderske, 2013; Verstraeten et al., 2009) which can compromise speech perception (Polonenko et al., 2016) and, on rare occasions, have been associated with skin necrosis (Chen et al., 2017). There have also been concerns around providing sufficient gain from passive transcutaneous BCDs in the context of mixed hearing loss (Mylanus et al., 2020).

By contrast, active transcutaneous devices use an external component which transmits electrical signals to the internal implant. These devices have reduced skin complications (Chan et al., 2017; Cushing et al., 2024; Stevens et al., 2025) and have better high frequency sound conduction (Stevens et al., 2025). One example is the Cochlear™ Osia^®^ (Osia) which contains a subcutaneous piezoelectric transducer to generate vibrations of the implant (Stevens et al., 2025; Vagle et al., 2024). The piezoelectric technology allows for bone conduction without significant surgical bone excavation needed by traditional electromagnetic transducers (Mylanus et al., 2020). Outcomes of Osia implantation in children and adolescents with conductive/mixed hearing loss include improved word recognition scores, speech-in-noise testing, sound localization, audiometric pure tone averages, and self-reported hearing (Florentine et al., 2022; Gordon et al., 2022; Magele et al., 2019; Stevens et al., 2025) with low rates of complication (Cushing et al., 2022).

Given the above considerations BCDs, particularly percutaneous ones, have traditionally been provided on one side even in children with bilateral conductive or mixed hearing loss (Priwin et al., 2007) despite what is known regarding the sensitive period for bilateral hearing in development (Gordon et al., 2015). Spatial hearing is an important function of binaural hearing and can be disrupted by hearing loss. This has been shown by acute unilateral ear attenuation using an earplug in listeners with typical hearing (Alemu et al., 2024b) as well as in adults and children with bilateral and unilateral hearing loss (Häusler et al., 1983; Noble et al., 1997). The degree and type of hearing loss are important considerations (Häusler et al., 1983; Noble et al., 1994, 1997). Severe impairments in the low (0.25–1 kHz) or mid (2–4 kHz) frequency ranges can considerably impair localization accuracy, particularly in the frontal horizontal plane (Häusler et al., 1983; Noble et al., 1994). Localization accuracy is also significantly reduced for listeners with asymmetric and unilateral losses compared to those with symmetrical losses (Häusler et al., 1983; Kim et al., 2017). These individuals show increased reliance on monaural spectral pinna cues (Agterberg et al., 2014). Conductive hearing loss typically produces only partial attenuation of acoustic input and remaining hearing may support spatial hearing including allowing reweighting of interaural cues in the case of asymmetric conductive loss (Keating & King, 2013). Yet, it is unclear whether this would occur in children with bilateral microtia given that they typically experience maximal conductive hearing loss and require BCDs to access sound for speech and language development (Chen et al., 2022; Fan et al., 2019; Kelley & Scholes, 2007). In these children, bilateral BCDs could be important for both development of language and spatial hearing (Gawliczek et al., 2018; Stenfelt & Zeitooni, 2013).

In adults with conductive hearing loss in both ears, bilateral BCDs have been shown to improve audiological thresholds for tones, speech recognition, quality of life, and sound localization relative to unilateral BCDs (Bosman et al., 2001; Ho et al., 2009; Janssen et al., 2012; Snik et al., 1998; Van Der Pouw et al., 1998). Increased risk of adverse events associated with surgery of two devices (Janssen et al., 2012) and additional costs (Colquitt et al., 2011) should be considered. On the other hand, there may be benefits of device redundancy (one device as back up for the other) (Durvasula et al., 2007; Janssen et al., 2012).

The use of bilateral BCDs for improved binaural/spatial hearing remains an open question. One concern is that the spatial hearing benefits of bilateral BCDs could be limited by transcranial cross-talk of bone conducted input to both cochleae (Snik et al., 1998) which would hinder access to the interaural cues needed for spatial hearing (McLeod et al., 2018). Transcranial attenuation of bone conduction stimuli falls anywhere from −5 to +20 dB between 250 Hz and 10 kHz with significant variability between individuals (Janssen et al., 2012). Moreover, transcranial attenuation and delay between the two ears can vary between individuals (Farrell et al., 2017). Results of bilateral BCDs in adults with hearing loss have been mixed with some studies showing benefits in sound localization over unilateral devices (Priwin et al., 2004; Snik et al., 1998) and other studies finding limited localization ability beyond lateralization of sound (Bosman et al., 2001; Caspers et al., 2022).

Effects of bilateral BCDs in children and adolescents on spatial hearing are not well-defined. Children with congenital atresia bilaterally have reportedly shown better spatial release of masking gains and improved sound localization (Chen et al., 2022; Den Besten et al., 2020; Dun et al., 2013; Priwin et al., 2007) with bilateral compared to unilateral BCDs. Still, unilateral BCDs remain more common than bilateral BCDs even in children with bilateral conductive hearing loss who cannot use traditional hearing aids. These limited studies also presented data from individuals using percutaneous BCDs with only one study reporting sound localization ability in bilateral active transcutaneous devices (i.e., MED-EL Bonebridge; Chen et al., 2022). The present study sought to expand these data by examining children receiving the Osia device and by assessing localization of both stationary and moving sound. It is possible that the improved high frequency sound conduction provided by the Osia (Stevens et al., 2025) supports better perception of interaural level cues, thereby improving sound localization.

Another novelty of the present study was that perception of moving sound was also examined as it appears to require complex auditory processing of binaural cues and is more challenging than localization of stationary sound (Harris, 1972; Middlebrooks & Green, 1991), particularly for individuals with hearing loss (Gessa et al., 2022; Klishova et al., 2021). Perception of movement direction and position can be reduced through acute unilateral ear plugging (Alemu et al., 2024b) and is particularly impaired in children who use bilateral cochlear implants (CIs) (Alemu et al., 2025) and bimodal hearing (Alemu et al., in review; Gordon et al., 2023). Impaired perception of sound in hearing loss in these cohorts was associated with poor access to interaural timing differences (ITDs) due to effects of early deafness (Tillein et al., 2016) and consequences of inaccurate binaural cues through the hearing devices (Kan et al., 2019). This was further shown by reduced ITD sensitivity and poor binaural fusion to bilateral input presented through headphones in children using bilateral hearing aids (Alemu et al., 2024a; Gorodensky et al., 2019).

In the present study, localization of both stationary and moving sound were measured with unrestricted head movements. Restriction of natural head and eye movements is common in sound localization studies because these movements support spatial hearing abilities (Brimijoin & Akeroyd, 2014; Pollack & Rose, 1967; Wallach, 1940; Wightman & Kistler, 1999). Unilateral ear plugging in listeners with typical hearing decreased sound localization accuracy but they demonstrated a distinct strategy of head movements that increased the signal to the exposed (nonplugged) ear (Alemu et al., 2024b). These movements were included in the present study as they may support spatial hearing in children with hearing loss. The same paradigm in children with hearing loss revealed that they made some attempts to use strategic head and eye movements to support impaired spatial hearing. Bilateral cochlear implant users demonstrated larger head movements to sounds further away from center and better localization of these more peripheral sound sources (Alemu et al., 2025). By contrast, bimodal listeners attempted to move their heads to expose their acoustic hearing ear to sounds presented on the contralateral side during sound localization tasks (Alemu et al., in review).

The objectives of the current study were to characterize the effects of bilateral BCDs on perception of stationary and moving sound in children and adolescents with bilateral microtia and/or atresia. The current study assessed spatial hearing in children using bilateral Osia devices or an Osia on one side and a Cochlear™ percutaneous Baha^®^ Connect System (Baha) on the other side.

The following hypotheses were tested: (1) bilateral devices improve speech perception in noise for spatially separated compared to co-located sound sources; (2) localization of stationary sound improves with bilateral versus unilateral BCD use but remains impaired relative to normal hearing; (3) perception of moving sound direction improves with bilateral versus unilateral BCD use; and (4) head movements of bilateral BCD users follow sound position or direction less than normal hearing.

## Methods

### Participants

This work was conducted with approval from the Research Ethics Board at the Hospital for Sick Children in Toronto, Canada. As detailed in Table 1, 11 children and adolescents [*M*_Age_(SD) = 14.7 (3.5) years; 4 F:7 M] with bilateral atresia/stenosis who had received surgical BCDs bilaterally participated. While all but one child had congenital atresia/stenosis, one subject developed acquired stenosis with myringitis. All participants received a Cochlear™ Osia^®^ (Osia) device; five had an Osia on one side with a Cochlear™ percutaneous Baha^®^ Connect System (Baha) on the opposite side and six had bilateral Osia devices. Many of the children also had microtia which could also affect their unaided spatial hearing at high frequencies.

**Table 1.** Demographic and Hearing Outcomes for 11 Bilateral BCD Users are Summarized Below.

No.	ID	Sex	Age at Test (Years)	BCD Configuration (Left/Right)	Age at Bil. BCD (Years)	Bil. BCD Experience (Years)	Etiology (Onset)	PTA 500, 1000, 2000 Hz (dB) [Left/Right]
1	P01	F	17	Baha/Osia	15.0	1.9	Treacher collins syndrome (congenital)	66.7/65.0
2	P02	M	11	Osia/Osia	10.9	0.05	Unknown (congenital)	63.3/61.7
3	P03	F	20	Osia/Osia	17.9	2.1	Chronic myringitis (acquired)	41.7/-
4	P04	F	14	Osia/Osia	13.2	0.8	Unknown (congenital)	48.3/35.0
5	P05	M	17	Baha/Osia	16.3	0.68	BOR syndrome (congenital)	80.0/68.3
6	P06	M	16	Baha/Osia	15.6	0.36	Nager syndrome (congenital)	75.0/68.3
7	P07	M	17	Osia/Baha	14.8	2.2	Unknown (congenital)	73.3/70.0
8	P08	M	16	Osia/Osia	14.6	1.4	Unknown (congenital)	68.3/71.7
9	P09	M	7	Osia/Osia	6.7	0.27	Unknown (congenital)	58.3/56.7
10	P10	M	13	Osia/Osia	12.2	0.81	Unknown (congenital)	60.0/65.0
11	P11	F	14	Osia/Baha	12.8	1.2	Unknown (congenital)	56.7/56.7

Of these children and adolescents, 6 wore bilateral Osia devices and 5 wore one Baha and an Osia on the contralateral ear. BCD: bone conduction device; PTA: pure tone average.

The BCD group was compared to a cohort of 11 age-matched children and adolescents with typical hearing (controls) [*M*_Age_(SD) = 14.9 (1.9) years; 4 M:7 F]. Audiometric data from the BCD group were available from medical records. Aided testing was performed with each device worn alone with no occlusion or masking of the opposite ear. Participants in the control group had hearing thresholds at frequencies from 250 Hz to 8 kHz of ≤20 dB HL. Sound localization data from these participants have been previously published (Alemu et al., 2024b, 2025, in review).

### Speech Perception Testing

Speech perception testing was conducted using Grason-Stadler (GSI) audiometers in double-walled sound booths calibrated to American National Standards Institute (ANSI) standards with results available from medical records. The BCD group completed the Phonetically Balanced Kindergarten (PBK) test (Haskins, 1949) as part of clinical follow up. Word recognition scores (%) were collected in left, right unilateral aided conditions (no occlusion or masking of the unaided ear) and bilaterally aided conditions in both quiet [60 dB Sound Pressure Level (SPL) from a speaker positioned 1 m from the child in front at 0° azimuth] and amidst co-located speech weighted noise [+10 dB speech-to-noise ratio (SNR)].

Speech reception thresholds (SRTs) were measured using a GSI audiometer in a double-walled sound booth calibrated to ANSI standards in the bilateral aided condition with speech presented from a loudspeaker 1 m in front (0° azimuth) using a bracketing procedure (American National Standards Institute, 2004) in 4 dB down 2 dB up steps amidst constant noise (45 dB HL) presented in three positions: co-located (S0°N0°), 90° leftward (S0°N-90°), and 90° rightward (S0°N + 90°). Conditions were tested in random order. Spatial release from masking (SRM) was calculated as the improvement in SRTs during spatial separation leftward or rightward relative to co-located presentation [*SRM*_(−90°)_ = *SRT*_(S0°N0°)_ – *SRT*_(S0°N−90°)_; *SRM*_(+90°)_ = *SRT*_(S0°N0°)_ – *SRT*_(S0°N_ _+_ _90°)_]. SRT testing was not completed in three children and adolescents who use BCDs due to time constraints but was completed in all participants in the control group.

### Self-Reported Hearing

The Speech, Spatial and Qualities of Hearing Scale (SSQ) was administered to characterize self-reported hearing challenges (Galvin & Noble, 2013; Gatehouse & Noble, 2004) in children and adolescents who use BCDs. An adolescent version was used in participants who could comprehend questions and respond independently and a parental version for parents/caregivers was used for younger participants who were unable to complete responses independently. The SSQ features hearing scenarios in which the respondent indicates a corresponding hearing ability using a 10-point numeric scale with a score of “10” representing perfect hearing and a score of “0” representing inability to hear. The SSQ includes questions describing every-day hearing situations across three thematic sections including speech, spatial hearing, and qualities of hearing. Responses to the SSQ were not available in two children and adolescents who use BCDs due to time constraints.

### Localization of Stationary and Moving Sound

Sound localization testing was completed in all participants in response to stationary and moving sound presentation in a cubic (2m×2m×2m) soundbooth. The tests were administered using an “L-shaped” moving arm setup as described in previous studies by our group (Alemu et al., 2024b, in review; Gordon et al., 2023). The L-shaped moving arm features a small loudspeaker fixed to the distal end of an L-shaped moveable arm operated by a 57BYGH420-2 Wantai silent-stepper motor which allows for presentation of stationary and moving sound anywhere in a frontal-horizontal (azimuthal) arc of 120° without the need for a speaker array or simulated movement. The motor and arm were visually obstructed from the participant by an acoustically transparent black curtain. The stimulus used was bandpass-filtered white noise (125Hz–8kHz). The location response was collected as continuous azimuthal position recorded at button press using a Logitech Gamepad F310 videogame controller.

For each trial, the sound first moved to an initial position (location 1; L1) and was presented stationary for three seconds. The sound was then presented while moving from L1 to a secondary location (location 2; L2). During the sound movement (L1-to-L2) component of the trial, sound would remain stationary or move by ±20° [*M*_Duration_(SD) = 6.7 (0.4) s; *M*_Velocity_ = 3.0 °/s] or ±40° [*M*_Duration_(SD) = 9.0 (0.2) s; *M*_Velocity_(SD) = 4.4 °/s]. Leftward and rightward movements were presented in equal number but in random order. All sound presentations were balanced between the left and right hemifields (0° to −60° and 0° to +60°, respectively) with no movements crossing the central midline (0°). Participants indicated “*Where is the sound?*” (stationary) and “*Where did the sound move to?*” (moving) by pointing a laser to the location using a videogame controller. Responses were collected for each presentation as well as time of the start of their responses (time between stimulus offset and start of response). Testing was administered in five to seven blocks of six trials per block for a total of 30 to 42 trials per condition (one stationary presentation and one moving presentation per trial). Testing was completed in three testing conditions in children and adolescents who use BCDs (i.e., left device only, right device only and bilateral devices on) in random order.

### Head and Eye (Gaze) Movement Collection

During sound localization concurrent unrestricted head and eye (gaze) movements were recorded using wearable technology. Head tracking data were available in 11 participants collected using the EDTracker Pro wireless head tracker which tracked movement at a rate of 125 samples per second along three degrees of freedom.

Head movements to the nearest tenth millisecond (∼0.01 s) were averaged for binned stationary sound locations in increments of 10°and for each movement condition (0°, ±20°, and ±40°) for moving sound localization).

### Statistical Analysis

All statistical analyses and graphical figures were generated using the R programming language for statistical computing and graphics (Version 4.0.3) and the R-Studio integrated development environment (Version 1.3.1093) (2021, 2020). Parametric statistical tests were used to test for effects of factors such as group and listening condition or stimulus condition on study outcomes. As most of the BCD had microtia, this factor could not be assessed. The primary statistical test used was mixed-effects linear regression analysis using type III analysis of variance (ANOVA) using Satterthwaite's method. Additional tests were used including two-sample t-tests and two-way ANOVA depending on the data structure and variables in question. For interpretation of perceived direction of sound movement, logistic regression analysis was used. Specifically, direction perception was calculated by converting errors in response to moving sound localization to a binary variable (“1” for rightward and “0” for leftward) which was used to model proportion of responses judged to be moving rightward. See Appendix A for details of specific statistical analyses.

## Results

**Figure 1.**
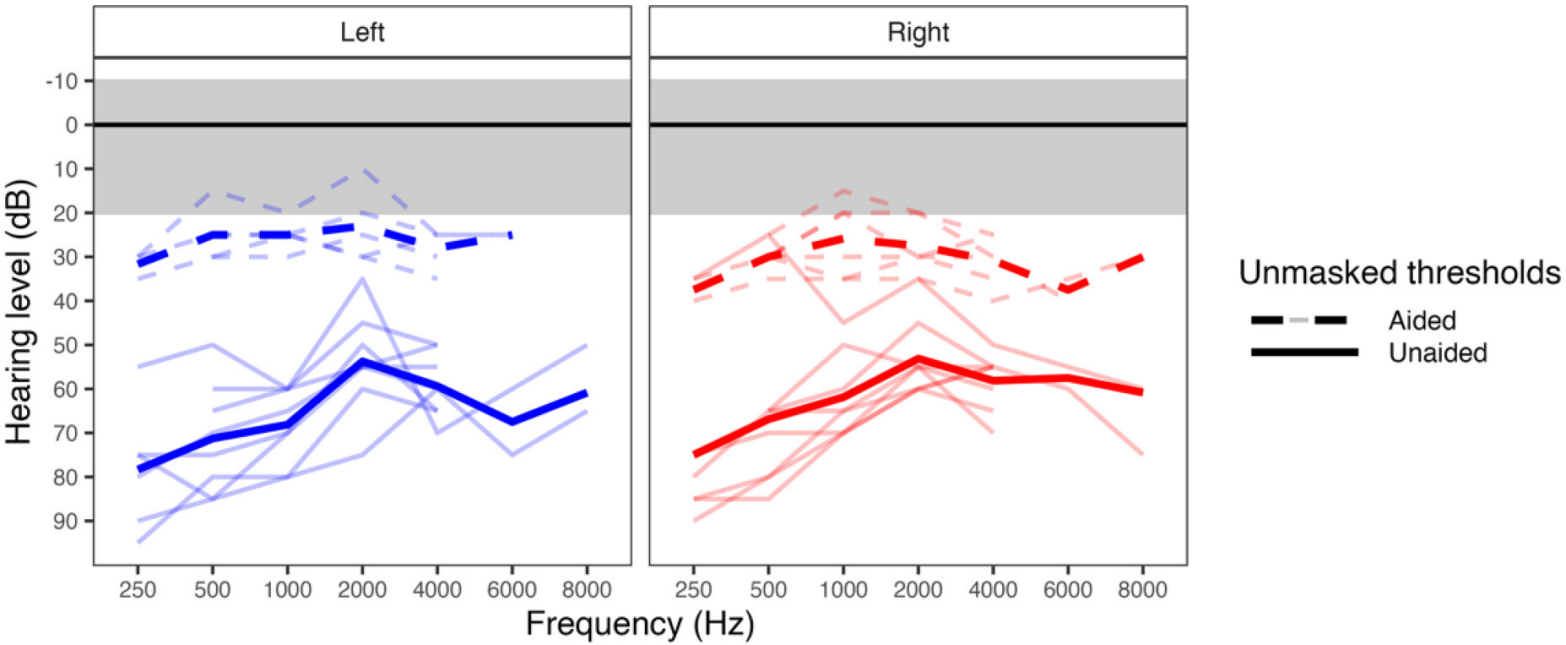
Audiograms in aided and unaided conditions are shown for 10 participants with bilateral bone conduction devices (BCDs). For all controls thresholds fell within the typical range of hearing (≤20 dB HL) which are not shown. Pure-tone averages calculated across 0.5, 1 and 2 kHz were significantly higher in children and adolescents who use BCDs compared to controls (*p* < .001) for unaided hearing levels, and overall thresholds were higher on the left side (*p* < .05) indicating a right ear bias across groups.

Audiometric hearing results are shown in Figure 1, thresholds revealed moderate to severe hearing loss bilaterally in the BCD group [*M*_(Left/PTA)_ (SD) = 64.4 (13.0) dB; *M*_(Right/PTA)_ (SD) = 60.6 (13.9) dB] with better aided audibility [*M*_(Left/PTA)_ (SD) = 24.3 (5.9) dB; *M*_(Right/PTA)_ (SD) = 27.8 (6.2) dB]. Thresholds were normal (≤20 dB HL) in the control group.

### Speech Perception Declines in Noise for Children and Adolescents Who Use BCDs During Unilateral Listening

**Figure 2.**
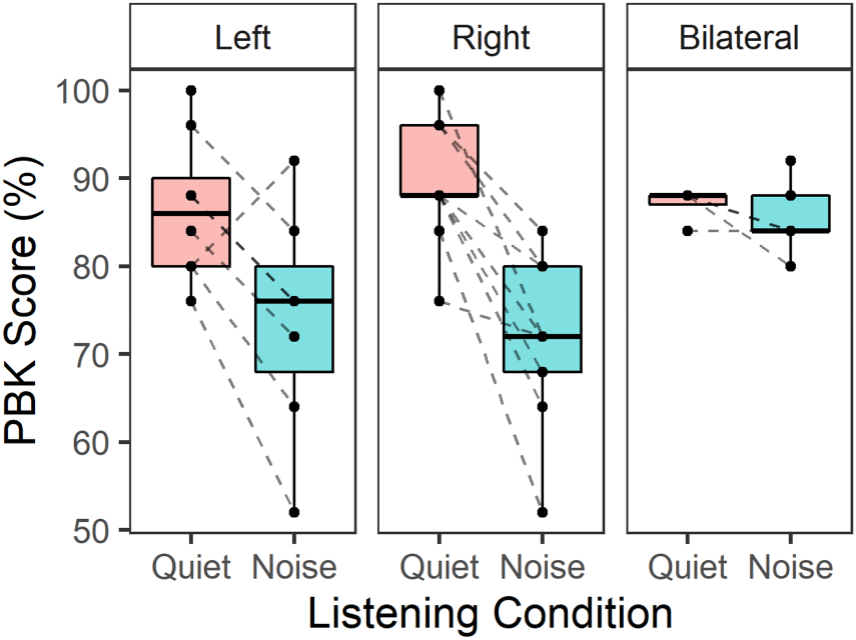
Phonetically Balanced Kindergarten (PBK) accuracy by listening condition (quiet and noise) and divided by ear of presentation (left unilateral, right unilateral, or bilateral). Dashed lines indicate individual participants.

Word recognition scores on the PBK test in children and adolescents who use BCDs are plotted in Figure 2 for nine participants. PBK scores were better in quiet than noise [Estimate (SE) = 11.6 (2.1) %, *p* < .001] and better with bilateral than either the left [Estimate (SE) = 7.4 (2.7) %, *p* < .05] or right [Estimate (SE) = 7.6 (2.7) %, *p* < .05] device alone. There was a just-significant interaction between device condition and noise condition [*F*_(2, 30)_ = 3.3, *p* = .05] reflecting smaller declines in noise with bilateral than unilateral devices in BCD participants. Limited data measured in the bilateral condition restricted statistical comparisons between this and the unilateral condition.

**Figure 3.**
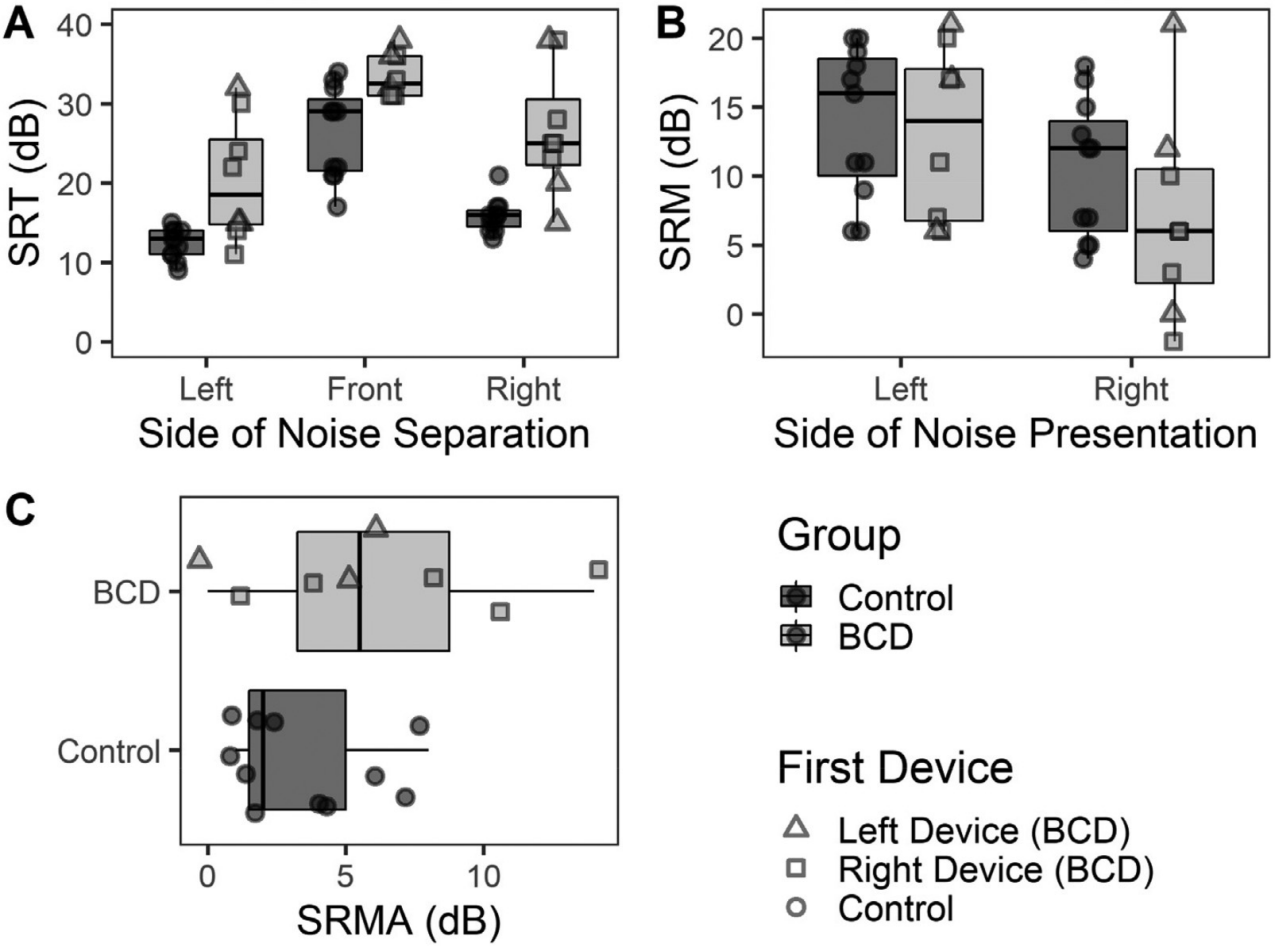
(A) SRTs were significantly higher in children with BCDs than controls during co-located presentation compared to spatially separated presentation (*p* < .001). (B) SRM was not significantly different in children and adolescents who use BCDs compared to controls (*p* = .29) (C) SRM asymmetry (SRMA) [dB] showed a right ear advantage and was similar in children and adolescents who use BCDs and controls (*p* = .18). BCD: bone conduction devices; SRM: spatial release from masking; SRMA: SRM asymmetry; SRT: speech reception threshold.

As shown in Figure 3A, SRTs were significantly higher in children and adolescents who use BCDs compared to age-matched controls [Estimate (SE) = 8.6 (1.9) dB, *p* < .001]. SRTs in response to co-located noise presentation were significantly higher than spatially separated noise presented on the left [Estimate (SE) = 13.5 (1.2) dB, *p* < .001] and right [Estimate (SE) = 8.7 (1.2), *p* < .001]. These benefits of spatial separation, known as SRM, are plotted in Figure 3B. SRM did not differ significantly in children and adolescents who use BCDs compared to controls [Estimate (SE) = 2.1 (2.7) dB, *p* = .43] and higher during noise presentation to the left side [Estimate (SE) = 4.8 (0.8) dB, *p* < .001], indicating a right ear bias in both groups. This asymmetry in the SRM, plotted in Figure 3C, was marginally higher in BCD compared to age-matched peers with typical hearing however the difference was not statistically significant [*t*_(9.9)_ = −1.4, *p* = .18].

### Children and Adolescents Who Use BCDs Self-Report Spatial Hearing Challenges

**Figure 4.**
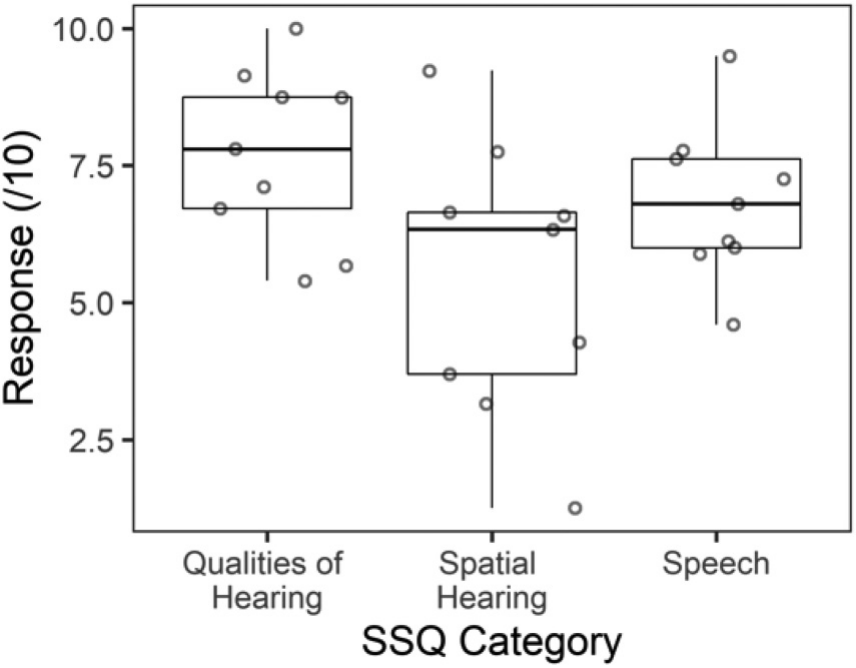
Self-reported hearing as measured by the Speech, Spatial and Qualities of Hearing Scale (SSQ) are shown for bilateral bone conductive device users with lowest scores on the spatial hearing subtest (*p* < .001).

Self-reported hearing measured by the SSQ was available in nine participants with BCDs. As shown in Figure 4, lowest scores occurred on the spatial hearing subtest [*F*_(2, 16)_ = 12.8, *p* < .001].

### BCDs Support Localization of Stationary Sound

**Figure 5.**
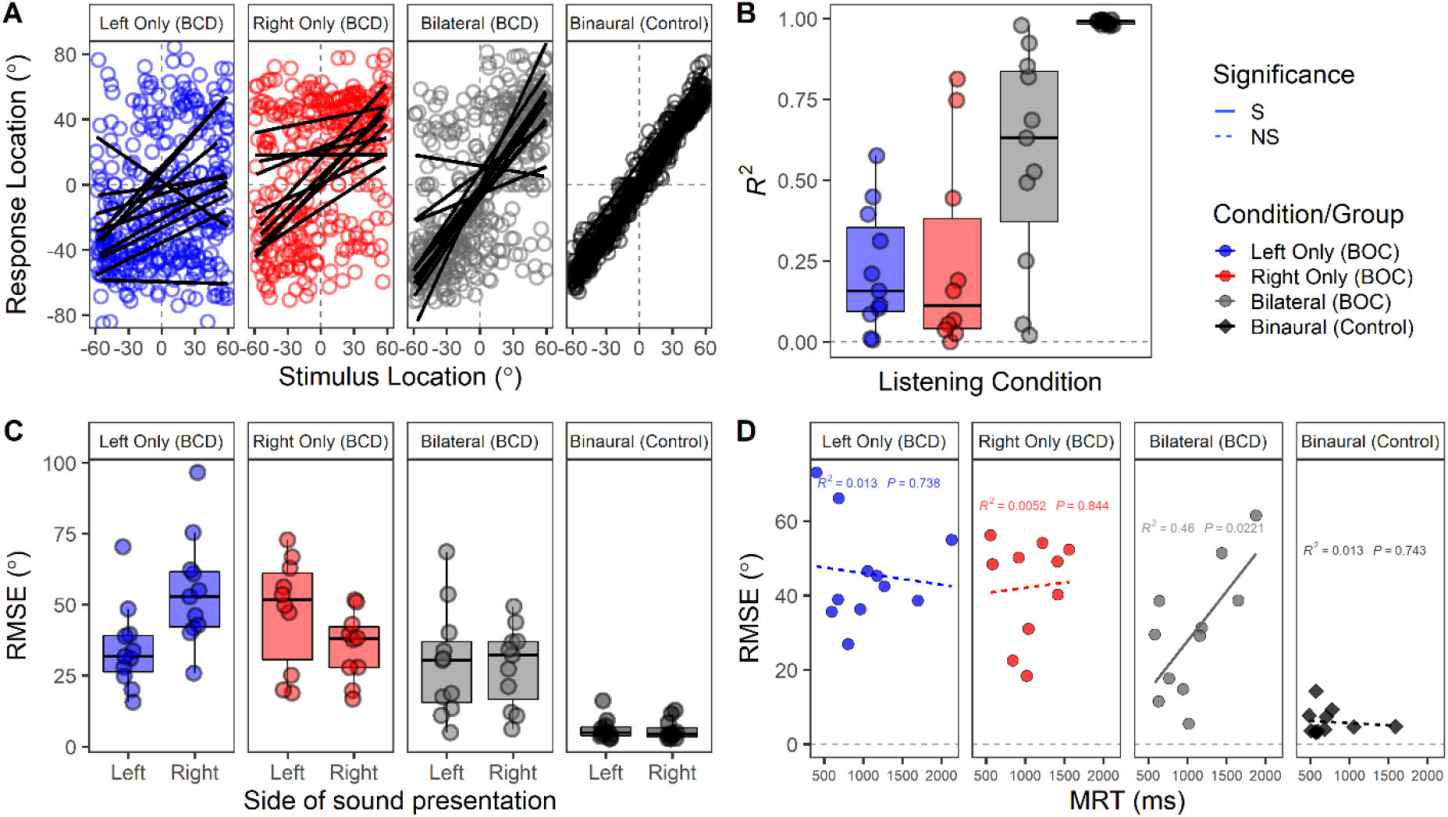
(A) Responses for all participants and trials against azimuthal sound position with simple linear regression curves shown by participant. (B) Coefficient of determination (*R*^2^) was reduced in bilateral bone conduction device (BCD) users compared to controls (*p* < .001) and when listening with one device compared to two (*p* < .05) [one outlier participant under the right device listening condition not visible]. (C) Root mean square error (RMSE) [°] by hemifield of sound presentation was higher in children and adolescents who use BCDs compared to controls (*p* < .001) and during listening with one device alone (*p* < .01). (D) RMSE (°) is plotted against mean reaction time (MRT) [ms]. Higher MRT was associated with higher error for children and adolescents who use BCDs when listening bilaterally (*p* < .05) but not one device alone (*p* > .05).

Responses to presentation of stationary sound between (−60° and +60°), were available in all 11 children and adolescents who use BCDs and 11 age-matched controls. As shown in Figure 5, the children and adolescents who use BCDs completed localization under three different device configurations: (i) both devices on, (ii) left device only (right device removed), and (iii) right device only (left device removed). Participant responses are plotted against sound azimuthal position across trials in Figure 5A. Response distributions were more dispersed and further from the line of unity (y = x) for children and adolescents who use BCDs compared to controls.

The coefficient of determination (*R*^2^) was extracted from individual simple linear regression models to demonstrate response reliability by participant in Figure 5B. *R*^2^ was significantly reduced for children and adolescents who use BCDs compared to controls across all device conditions [*F*_(1, 36.1)_ = 17.0, *p* < .001]. For children and adolescents who use BCDs, *R*^2^ values improved during bilateral listening compared to left [Estimate (SE) = 0.35 (0.09), *p* < .01] and right unilateral listening [Estimate (SE) = 0.33 (0.09), *p* < .01].

Root mean square error (RMSE) [°] by hemifield of sound presentation is plotted in Figure 5C. RMSE was significantly higher in children and adolescents who use BCDs compared to controls [*F*_(1, 43.4)_ = 65.1, *p* < .001] and in the BCD group, RMSE was significantly reduced when listening bilaterally compared to the left [Estimate (SE) = 15.8 (3.9)°, *p* < .001] or right device alone [Estimate (SE) = 13.4 (4.0)°, *p* < .01]. There was a significant interaction between device condition and hemifield [*F*_(2, 63)_ = 8.5, *p* < .001], reflecting increased error on the side of the nonaided ear in unilateral conditions and more symmetric responses in the bilateral conditions. Finally, as shown in Figure 5D, response accuracy was lower in participants with BCDs than controls [*F*_(1, 46.4)_ = 50.3, *p* < .001]. Their mean response times increased with increasing errors when listening with both devices (*p* < .05) but remained consistently high in unilateral conditions (*p* > .05).

### Perception of Moving Sound Impaired in Children and Adolescents Who Use BCDs

**Figure 6.**
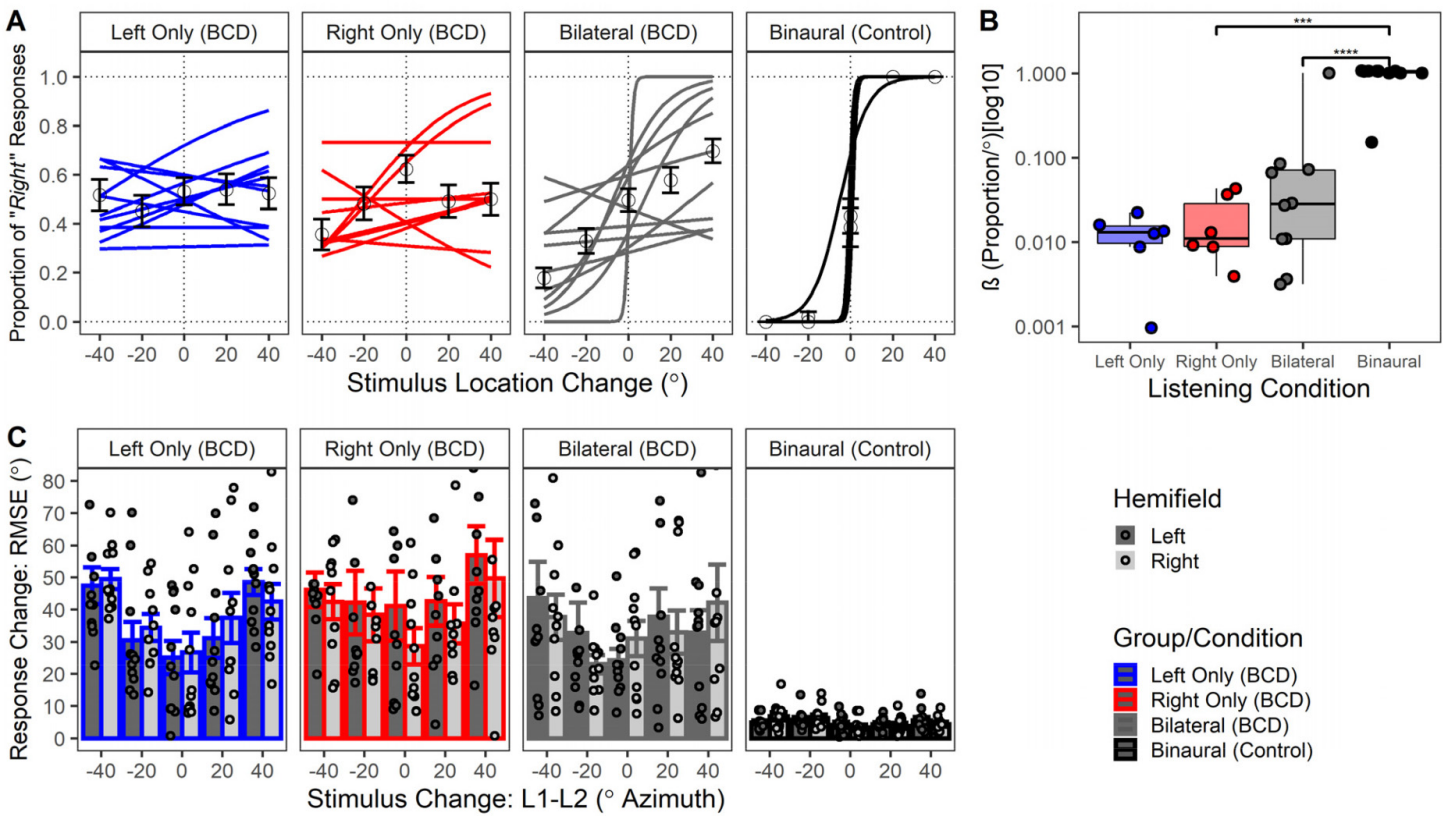
(A) Perception of moving sound direction using logistic regression curves plotted by stimulus location change (°). Logistic regression curves were positively sloping with a “sharp sigmoid” response pattern in controls unlike children and adolescents who use bone conduction devices (BCDs) whose response curves had flatter (sometimes negative) slopes. (B) Gaussian slopes (*β*) [proportion/°] were significantly reduced in children and adolescents who use BCDs compared to controls (*p* < .001). (C) Root mean square error (RMSE) [°] of the response change was significantly higher in children and adolescents who use BCDs compared to controls (*p* < .001).

Responses to presentation of moving sound (0°, ±20°, and ±40°) were available in all participants and across device listening conditions. Errors were converted to a binary variable representing perceived directionality where “1” represents response judgment rightward and “0” represents judgment leftward. The binary response was then modeled using logistic regressions per participant and listening condition. The logistic regression curves representing proportion of “right” responses are shown in Figure 6A. Responses in the control group were typically positively sloping sigmoidal curve patterns representing accurate direction perception. Gaussian slopes (*β*) were extracted from individual logistic regressions [logistic regression model given by: $log\left(\frac{P}{1-P}\right)+Intercept+\beta *X$ ] in both groups as shown in Figure 6B with a logarithmic [log_(10)_] transformation applied. Children and adolescents who use BCDs had significantly reduced *β* compared to controls [Estimate (SE) = 0.93 (0.07) proportion/°, *p* < .001]. As shown in Figure 6C, children and adolescents who use BCDs had significantly higher errors (RMSE of response change °) compared to controls [Estimate (SE) = 33.1 (4.1)°, *p* < .001]. Children and adolescents who use BCDs had significantly reduced RMSE when listening bilaterally compared to listening with only the right device [Estimate (SE) = 8.9 (2.5)°, *p* < .01] however the difference was not statistically significant between the bilateral and left device conditions [Estimate (SE) = 3.5 (2.4)°, *p* = .46].

### Children and Adolescents Who Use BCDs Have Abnormal Head Movements During Sound Localization

**Figure 7.**
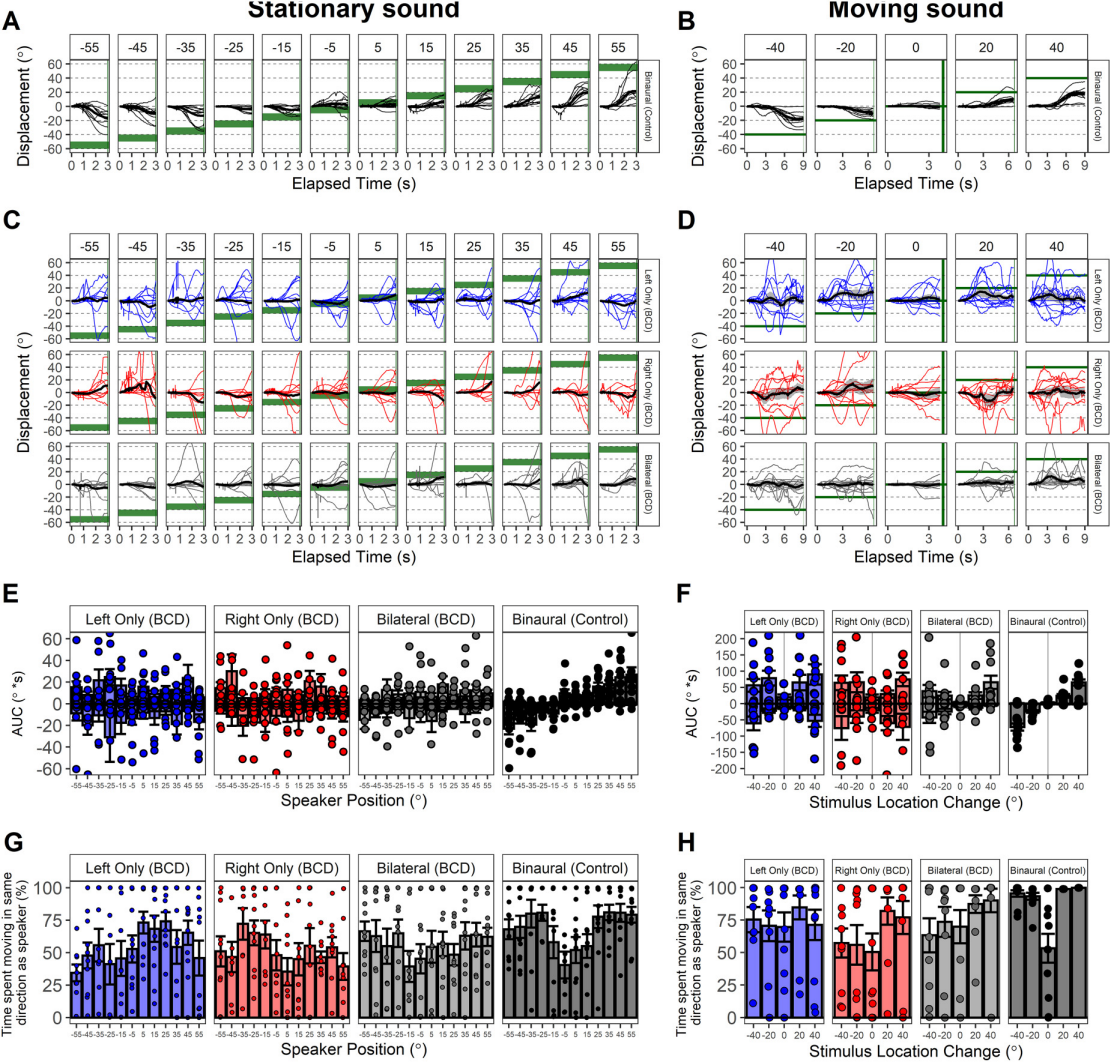
Head displacement (°) waveforms are shown in response to stationary (A, C) and moving (B, D) sound presentation. Waveforms were summarized as area under the curve (AUC)[°*s] in response to stationary (E) and moving (F) sound presentation. AUC was significantly higher for children and adolescents who use bone conduction devices (BCDs) compared to controls across tasks (*p* < .05). Proportion of time spent moving in correct hemifield/direction (%) was quantified for responses to stationary (G) and moving (H) sound presentation. Proportion time spent moving in correct direction was significantly reduced in children and adolescents who use BCDs (*p* < .05).

Head displacement waveforms are plotted by participant in response to stationary (Figure 7A and C) and moving (Figure 7B and D) sound presentations. In the control group, head displacement follows sound position in direction and magnitude for both stationary (Figure 7A) and moving sound (Figure 7B). The waveforms in the children and adolescents who use BCDs indicate more random or uncertain movements (Figure 7C and D). Head displacement was summarized as area under the curve (AUC) [°*s] for stationary (Figure 7E) and moving (Figure 7F) sound presentations. AUC was significantly higher for children and adolescents who use BCDs compared to controls for stationary [Estimate (SE) = 4.1 (2.9) °*s, *p* = .18] and moving [Estimate (SE) = 22.1 (8.2) °*s, *p* < .05] sound presentations. Head displacement was also measured as the proportion of time spent moving in the same direction as the speaker (%) for stationary (Figure 7G) and moving (Figure 7H) sound presentations. The proportion of time spent moving in the same direction was significantly lower for children and adolescents who use BCDs compared to controls for stationary [Estimate (SE) = −14.1 (3.6) %, *p* < .001] and moving [Estimate (SE) = −16.3 (5.1) %, *p* < .01] sound presentations.

## Discussion

The present study explored spatial hearing abilities in children and adolescents fitted with bilateral BCDs, specifically users provided either with Cochlear™ Osia^®^ (Osia) devices bilaterally or a single Osia with a percutaneous Cochlear™ Baha^®^ Connect System (Baha) on the contralateral side, compared to unilateral device use. The study provided support for two of the four hypotheses. Benefits of bilateral device use compared to unilateral device were found for speech perception in noise and for localization of stationary sound sources. Remaining impairments in perception of moving sound direction and abnormal head movements during sound localization tasks suggest that binaural processing remains challenging to provide through bilateral BCDs. These findings are clinically relevant as the decision to provide bilateral implants should be weighed against risk of adverse events associated with surgery and additional costs.

### BCDs Improve Speech-in-Noise

Children and adolescents who use BCDs demonstrated fairly good word recognition scores in quiet across all listening conditions (Figure 2). Scores were not perfect which reflects effects of hearing loss on speech perception and limited receptive and expressive language skills in children with hearing loss. Accuracy declined significantly in noise during unilateral listening. Children and adolescents who use BCDs had higher SRTs (poorer performance) in response to spondees in noise compared to peers with typical hearing but demonstrated similar benefit of spatial separation or SRM (see Figure 3). SRM benefits in children with bilateral BCDs in prior reports have been more modest than the present findings (Dun et al., 2013; Stenfelt & Zeitooni, 2013), potentially reflecting differences in task, stimuli, transducers, and noise. It is also noteworthy that children and adolescents who use BCDs had similar SRM benefit to peers with typical hearing [*M*_SRM_ (SD) = 10.5 (5.1) dB] which could be driven by head shadow for each ear rather than binaural processing. SRM also revealed a right-ear bias which has been reported both in controls and other cohorts of children with hearing loss in the same setup (Alemu et al., 2025, in review; Gordon et al., 2023). These data were collected in the same soundbooth calibrated to ANSI standards but the findings should be replicated in other settings. If the right-ear bias is a reflection of normal bilateral hearing, it may suggest that bilateral devices can mitigate the abnormal aural preference seen in children with asymmetric hearing loss (Gordon et al., 2015).

### Bilateral BCDs Improve Localization of Stationary Sound

Children and adolescents who use BCDs demonstrated ability to localize stationary broadband stimulus (see Figure 5). Response accuracy in the present sound localization paradigm were similar to data previously reported in children with normal hearing with and without a monaural plug and in children with hearing loss (Alemu et al., 2024b, 2025, in review; Gordon et al., 2023). Improved stationary sound localization in the BCDs when using bilateral rather than unilateral devices also demonstrates that transcranial attenuation was not negligible. They were able to utilize some binaural cues and may have partially resolved the head shadow as previously shown (Farrell et al., 2017; Stenfelt, 2012). This is also consistent with prior studies demonstrating benefits of bilateral BCDs in children and adults (Bosman et al., 2001; Chen et al., 2022; Gawliczek et al., 2018; Priwin et al., 2004, 2007; Zheng et al., 2022).

There are some signs that the Osia provides specific benefits. The three participants who had the lowest sound localization errors (around 10°–20°) were all bilateral Osia users. Further, listeners with a mixed device configuration (Osia on one side, and BAHA on the other) tended to have lower errors during unilateral listening with the Osia alone [*M*_RMSE_ (SD) = 36.1 (12.4)°] compared with the BAHA alone [*M*_RMSE_ (SD) = 40.9 (10.5)°] but this could also be interpreted as the mismatch between bilateral devices. Regardless of the type of BCD, unilateral BCD use created challenges for sound localization with higher errors on the unaided side (Figure 5). This reflects the hearing asymmetry as also seen in children and adolescents with asymmetric hearing using a CI in one ear and a hearing aid in the other (bimodal hearing) (Alemu et al., in review; Gordon et al., 2023) and in adolescents and adults with typical hearing fitted with a monaural ear plug (Alemu, 2020; Alemu et al., 2024b).

Variability in sound localization remained. Reaction time increased with larger response error (see Figure 5D), highlighting more effortful and less accurate responses in some participants. This variability could reflect individual variability in transcranial attenuation and delay (Farrell et al., 2017; Janssen et al., 2012). Further, despite the benefits of bilateral BCDs measured, the participants reported that spatial hearing remained challenging as measured by the SSQ (see Figure 4).

### Bilateral BCDs Do Not Support Perception of Moving Sound

This study is the first to the authors’ knowledge to measure perception of moving sound in children and adolescents who use BCDs. Children and adolescents who use BCDs demonstrated challenges perceiving direction perception in all conditions (Figure 6A and B) compared to the control group with only a slight improvement in error localizing the position sound moved to (L2) when using both devices compared to the left one (see Figure 6). This finding is similar to past work which found significant impairments in perception of movement direction in listeners with asymmetric hearing (Alemu et al., in review; Gordon et al., 2023) and in listeners with typical hearing who performed the task with acute monaural attenuation through an ear plug (Alemu et al., 2024b). Thus, while BCDs may alleviate the head shadow on the side of the device (Janssen et al., 2012) and provide bilateral hearing, these benefits do not appear to extend into the binaural processing needed to track moving sound or to determine moving sound direction (Figure 6C). Unlike prior cohorts, the present group of children and adolescents with hearing loss had conductive hearing loss and retained normal cochlear function. This suggests that the bilateral BCDs may not be providing sufficiently accurate interaural cues to enable this important aspect of binaural hearing. Bilateral BCDs, like bilateral CIs, and bilateral hearing aids, are independent devices that are programmed independently with limited ability to provide balanced interaural level differences and consistent interaural timing differences (Agterberg et al., 2011; McLeod et al., 2018). Each BCD has the added disadvantage of cross-talk to both cochleae with unclear effects when used bilaterally or bimodally (Agterberg et al., 2011; Chen et al., 2022).

### Children and Adolescents Who Use BCDs Have Ineffective Head Movements

Participants in the control group moved their heads toward the sound source with accurate direction and magnitude both for stationary (Figure 7A) and moving (Figure 7B) sound presentations, whereas children with BCD moved their heads differently. Findings in the control group are consistent with previously published gaze responses which demonstrated that both children and adults with typical hearing accurately follow sound with gaze during binaural listening (Alemu et al., 2024b). In children and adolescents with BCDs, head movements were larger than those made by the control group and were not related to the sound position for either stationary (Figure 7E) or moving (Figure 7F) sound. The increased movements recorded in the bilateral BCD group were not reflective of a gaze strategy in children with simulated unilateral conductive hearing loss (unilateral ear plug) who moved their heads to favor their unexposed better-hearing ear (Alemu et al., 2024b). Rather, increased head movements may reflect a behavioral response to a challenging hearing task and may be unrelated to spatial hearing. In support, this group reported hearing challenges in speech as well as spatial hearing domains (Figure 4). In a secondary analysis it was found that children and adolescents who use BCDs spent less time moving their head toward the correct direction or side of sound presentation compared to control participants both for stationary (Figure 7G) and moving (Figure 7H) sound. This was similar to previously studied cohorts who completed the same test paradigm (Alemu et al., 2025, in review). Noteworthy is that the bilateral CI users had reduced gaze in the correct direction over a particular range whereas the children and adolescents who use BCDs had a more general reduction (Alemu et al., 2025). This may reflect a more pronounced spatial hearing deficit associated with conductive hearing loss compared to sensorineural loss (Häusler et al., 1983) perhaps related to poor access to low-frequency interaural timing cues (Gorodensky et al., 2019; Noble et al., 1994).

### Study Limitations and Future Directions

The current study explored spatial hearing abilities in children and adolescents provided with surgical BCDs bilaterally. Spatial hearing was measured as ability to localize stationary and moving sound in the frontal-horizontal (azimuthal) plane using a moving arm setup which eliminated the need for speaker arrays and simulated sound movement. Though current findings support some availability of interaural cues, it is unclear to what extent these participants were able to access these cues. Hence, future work should measure sensitivity of these cues using the bone conduction modality. Further, positions beyond ±60° and at various elevations were not explored. Elevation variation would help to explore the role of potential monaural pinna cues which have previously been shown to affect horizontal localization in conductive hearing loss (Agterberg et al., 2012). Hence, future studies should also investigate perception of stationary and moving sound in a paradigm which allows presentation of sounds at positions beyond ±60° and at various elevations. Effects of daily device use should also be explored given known variability in children using hearing aids or cochlear implants (Gao et al., 2025; Walker et al., 2015) and potential for one device to be used more than the other (Gao et al., 2025). Lastly, in the present study eye tracking was not available due to poor suitability of the tracking equipment for the patient cohort. Therefore, future work should explore more robust wearable technology options which are better able to capture data of sufficient quality for exploration of composite gaze (head and eye summated) movements during localization of stationary and moving sound and could explore effects of training to support spatial hearing.

## Conclusion

Children and adolescents provided with bilateral bone conduction devices (Osia and percutaneous Baha devices) demonstrate improved perception of stationary sound and subtle improvements for perception of moving sound direction compared to using one device alone. These benefits indicate that they are able to take advantage of a reduction of the head shadow effect on each side and suggest that transcranial attenuation is not negligible. They also benefit from spatial release of masking but report ongoing hearing challenges.

## Appendix A

Tables below correspond to parametric statistical models shown in results by subsection and associated figure(s). Linear mixed-effects regressions are summarized using type III analysis of variance (ANOVA) using Satterthwaite's method.

### Table A1: Audiograms (Figure 1)

Linear mixed-effects model: PTA ∼ Group*Side + (1|IDs)

Unaided audiometric pure tone average (PTA) [dB] calculated across 500, 1k, 2k Hz

Group (2 levels) ∼ Control, BCD

Side of presentation (2 levels) ∼ Left, Right[Table table2-23312165261422955]

**Table table2-23312165261422955:**

Factor	Sum Sq	Mean Sq	NumDF	DenDF	F Value	Pr(>F)
Group	1920.597	1920.597	1	16.99999	254.8933	1.15E-11
Side	50.26914	50.26914	1	17	6.671502	0.019351
Group:Side	18.69019	18.69019	1	17	2.480481	0.133693

### Table A2: Speech perception declines in noise for BCD users during unilateral listening (Figure 2)

Linear mixed-effects model: PBK score ∼ Listening condition*Ear + (1|IDs)

Word recognition score on the Phonetically Balanced Kindergarten (PBK) test (%)

Listening condition (2 levels) ∼ Quiet, Noise

Ear (3 levels) ∼ Left unilateral, Right unilateral, Bilateral[Table table3-23312165261422955]

**Table table3-23312165261422955:**

Factor	Sum Sq	Mean Sq	NumDF	DenDF	F Value	Pr(>F)
Condition	1344.67	1344.67	1	30.5767	31.91717	3.49E-06
Ear	408.2718	204.1359	2	31.1241	4.84538	0.014724
Condition:Ear	275.6	137.8	2	30.04005	3.270828	0.051861

### Table A3: Spatial release from masking (Figure 3)

Linear mixed-effects model: SRT ∼ Group*Listening condition + (1|IDs)

Speech reception threshold (SRT) [dB]

Group (2 levels) ∼ Control, BCD

Listening condition (3 levels) ∼ Noise front (co-located), Noise left (separation −90°), Noise right (separation +90°)[Table table4-23312165261422955]

**Table table4-23312165261422955:**

Factor	Sum Sq	Mean Sq	NumDF	DenDF	F Value	Pr(>F)
Group	281.6044	281.6044	1	17	19.97315	0.000337
Condition	1740.348	870.174	2	34	61.71818	4.83E-12
Group:Condition	30.38317	15.19159	2	34	1.077482	0.351793

Linear mixed-effects model: SRM ∼ Group*Listening condition + (1|IDs)

Spatial release from masking (SRM) [dB]

Group (2 levels) ∼ Control, BCD

Listening condition (2 levels) ∼ Noise left, Noise right[Table table5-23312165261422955]

**Table table5-23312165261422955:**

Factor	Sum Sq	Mean Sq	NumDF	DenDF	F Value	Pr(>F)
Group	4.218288	4.218288	1	17	0.641415	0.43425
Condition	212.5147	212.5147	1	17	32.31406	2.69E-05
Group:Condition	16.51465	16.51465	1	17	2.511147	0.131468

### Table A4: BCD users self-report spatial hearing challenges (Figure 4)

Linear mixed-effects model: SSQ score ∼ Subtest + (1|IDs)

Speech, Spatial and Qualities of Hearing Scale (SSQ) score (/10)

Subtest (3 levels) ∼ Speech, Spatial hearing, Qualities of hearing[Table table6-23312165261422955]

**Table table6-23312165261422955:**

Factor	Sum Sq	Mean Sq	NumDF	DenDF	F Value	Pr(>F)
Subtest	23.61775	11.80887	2	16	12.8178	0.000476

### Table A5: BCDs support localization of stationary sound (Figure 5)

Linear mixed-effects model: *R*^2^ ∼ Group*Listening condition + (1|IDs)

Coefficient of determination (*R*^2^)

Group (2 levels) ∼ Control, BCD

Listening condition (4 levels) ∼ Binaural (Control), Left only (BCD), Right only (BCD), Bilateral (BCD)[Table table7-23312165261422955]

**Table table7-23312165261422955:**

Factor	Sum Sq	Mean Sq	NumDF	DenDF	F Value
Group	0.679412	0.679412	1	36.12579	16.9779
Condition	0.831018	0.415509	2	25.40372	10.38321
Group:Condition	#N/A	#N/A	#N/A	#N/A	#N/A

Linear mixed-effects model: RMSE ∼ Group*Listening condition + (1|IDs)

Root mean square error (RMSE) [°]

Group (2 levels) ∼ Control, BCD

Listening condition (4 levels) ∼ Binaural (Control), Left only (BCD), Right only (BCD), Bilateral (BCD)[Table table8-23312165261422955]

**Table table8-23312165261422955:**

Factor	Sum Sq	Mean Sq	NumDF	DenDF	F Value	Pr(>F)
Group	10570.69	10570.69	1	43.43427	65.05254	3.56E-10
Condition	3153.183	1576.592	2	64.24271	9.702418	0.000208
Side	36.04254	36.04254	1	63.52619	0.221807	0.639279
Group:Side	1109.557	1109.557	1	63.52619	6.828263	0.011188
Condition:Side	2756.55	1378.275	2	63.52619	8.481969	0.000543
Group:Condition	#N/A	#N/A	#N/A	#N/A	#N/A	#N/A
Group:Condition:Side	#N/A	#N/A	#N/A	#N/A	#N/A	#N/A

Linear mixed-effects model: RMSE ∼ MRT + Group*Listening condition + (1|IDs)

Root mean square error (RMSE) [°]

Mean reaction time (MRT) [ms]

Group (2 levels) ∼ Control, BCD

Listening condition (4 levels) ∼ Binaural (Control), Left only (BCD), Right only (BCD), Bilateral (BCD)[Table table9-23312165261422955]

**Table table9-23312165261422955:**

Factor	Sum Sq	Mean Sq	NumDF	DenDF	F Value	Pr(>F)
MRT	364.837	364.837	1	44.25639	1.889089	0.176225
Group	9633.783	9633.783	1	46.50996	49.88276	6.93E-09
Condition	3172.643	1586.321	2	69.19031	8.213812	0.00063
Group:Condition	#N/A	#N/A	#N/A	#N/A	#N/A	#N/A

Simple linear regression: RMSE ∼ MRT (Binaural-Controls)

Root mean square error (RMSE) [°]

Mean reaction time (MRT) [ms][Table table10-23312165261422955]

**Table table10-23312165261422955:**

Factor	Df	Sum Sq	Mean Sq	F Value	Pr(>F)
MRT	1	1.489659	1.489659	0.114068	0.743304
Residuals	9	117.5347	13.05941	#N/A	#N/A

Simple linear regression: RMSE ∼ MRT (BCD-Bilateral)

Root mean square error (RMSE) [°]

Mean reaction time (MRT) [ms][Table table11-23312165261422955]

**Table table11-23312165261422955:**

Factor	Df	Sum Sq	Mean Sq	F Value	Pr(>F)
MRT	1	1339.716	1339.716	7.624931	0.022064
Residuals	9	1581.319	175.7021	#N/A	#N/A

Simple linear regression: RMSE ∼ MRT (BCD-Left only)

Root mean square error (RMSE) [°]

Mean reaction time (MRT) [ms][Table table12-23312165261422955]

**Table table12-23312165261422955:**

Factor	Df	Sum Sq	Mean Sq	F Value	Pr(>F)
MRT	1	24.85785	24.85785	0.119186	0.73785
Residuals	9	1877.066	208.5629	#N/A	#N/A

Simple linear regression: RMSE ∼ MRT (BCD-Right only)

Root mean square error (RMSE) [°]

Mean reaction time (MRT) [ms][Table table13-23312165261422955]

**Table table13-23312165261422955:**

Factor	Df	Sum Sq	Mean Sq	F Value	Pr(>F)
MRT	1	8.638906	8.638906	0.041497	0.843668
Residuals	8	1665.455	208.1819	#N/A	#N/A

### Table A6: Perception of moving sound impaired in BCD users (Figure 6)

Linear mixed-effects model: *β* ∼ Group*Listening condition + (1|IDs)

Direction perception slope (*β*) [proportion/°]

Group (2 levels) ∼ Control, BCD

Listening condition (4 levels) ∼ Binaural (Control), Left only (BCD), Right only (BCD), Bilateral (BCD)[Table table14-23312165261422955]

**Table table14-23312165261422955:**

Factor	Sum Sq	Mean Sq	NumDF	DenDF	F Value	Pr(>F)
Group	4.759366	4.759366	1	38.37186	122.7933	1.6E-13
Condition	0.092084	0.046042	2	15.57681	1.1879	0.331018
Group:Condition	#N/A	#N/A	#N/A	#N/A	#N/A	#N/A

Linear mixed-effects model: RMSE ∼ Group*Listening condition*Side of presentation*Position change + (1|IDs)

Root mean square error (RMSE) [°]

Group (2 levels) ∼ Control, BCD

Listening condition (4 levels) ∼ Binaural (Control), Left only (BCD), Right only (BCD), Bilateral (BCD)

Side of presentation (2 levels) ∼ Left hemifield, Right hemifield

Position change (5 levels) ∼ 0°, ±20°, ±40°[Table table15-23312165261422955]

**Table table15-23312165261422955:**

Factor	Sum Sq	Mean Sq	NumDF	DenDF	F Value	Pr(>F)
Group	17388.87	17388.87	1	30.47087	55.70947	2.32E-08
Condition	4070.419	2035.209	2	369.2776	6.520289	0.001649
Position change	6981.1	1745.275	4	367.5896	5.591413	0.000223
Side	155.4894	155.4894	1	367.6716	0.498148	0.480762
Group:Position change	3434.277	858.5692	4	367.5565	2.750636	0.028059
Condition:Position change	2201.219	275.1524	8	367.5785	0.881518	0.532093
Group:Side	37.3528	37.3528	1	367.6021	0.119669	0.729592
Condition:Side	939.1788	469.5894	2	367.679	1.504444	0.223505
Position change:Side	7.254591	1.813648	4	367.5896	0.00581	0.999933
Group:Position change:Side	216.6439	54.16098	4	367.5565	0.173518	0.951907
Condition:Position change:Side	2240.09	280.0113	8	367.5785	0.897084	0.518908
Group:Condition	#N/A	#N/A	#N/A	#N/A	#N/A	#N/A
Group:Condition:Position change	#N/A	#N/A	#N/A	#N/A	#N/A	#N/A
Group:Condition:Side	#N/A	#N/A	#N/A	#N/A	#N/A	#N/A
Group:Condition:Position change:Side	#N/A	#N/A	#N/A	#N/A	#N/A	#N/A

### Table A7: Abnormal head movements do not facilitate accurate sound localization (Figure 7)

Linear mixed-effects model: AUC (L1) ∼ Group*Listening condition*Binned speaker position*Direction of movement + (1|IDs)

Area under the curve (AUC) [°*s] during stationary sound localization (L1)

Group (2 levels) ∼ Control, BCD

Listening condition (4 levels) ∼ Binaural (Control), Left only (BCD), Right only (BCD), Bilateral (BCD)

Binned speaker position (12 levels) ∼ −60° to (−50°), …, 50° to 60°

Direction of movement (2 levels) ∼ Leftward, Rightward[Table table16-23312165261422955]

**Table table16-23312165261422955:**

Factor	Sum Sq	Mean Sq	NumDF	DenDF	F Value	Pr(>F)
Group	1181.412	1181.412	1	26.43215	4.333969	0.047178
Condition	4263.734	2131.867	2	783.9462	7.82068	0.000433
Speaker Position	1079.864	98.16941	11	778.4121	0.360131	0.970666
Direction	510.599	510.599	1	778.0351	1.873115	0.171514
Group:Speaker Position	4288.821	389.8928	11	778.4435	1.430309	0.153979
Condition:Speaker Position	6289.784	285.8993	22	777.4656	1.048812	0.400088
Group:Direction	106.6211	106.6211	1	778.0286	0.391136	0.531886
Condition:Direction	8.395625	4.197813	2	777.3445	0.0154	0.984719
Speaker Position:Direction	9863.153	896.6503	11	778.0802	3.289331	0.000199
Group:Speaker Position:Direction	8454.777	768.6161	11	778.0836	2.819642	0.00128
Condition:Speaker Position:Direction	9747.207	443.0548	22	777.2757	1.625332	0.035121
Group:Condition	#N/A	#N/A	#N/A	#N/A	#N/A	#N/A
Group:Condition:Speaker Position	#N/A	#N/A	#N/A	#N/A	#N/A	#N/A
Group:Condition:Direction	#N/A	#N/A	#N/A	#N/A	#N/A	#N/A
Group:Condition:Speaker Position:Direction	#N/A	#N/A	#N/A	#N/A	#N/A	#N/A

Linear mixed-effects model: AUC (L1-to-L2) ∼ Group*Listening condition*Direction of movement*Position change + (1|IDs)

Area under the curve (AUC) during moving sound localization (L1-to-L2)

Group (2 levels) ∼ Control, BCD

Listening condition (4 levels) ∼ Binaural (Control), Left only (BCD), Right only (BCD), Bilateral (BCD)

Side of presentation (2 levels) ∼ Left hemifield, Right hemifield

Position change (5 levels) ∼ 0°, ±20°, ±40°

Direction of movement (2 levels) ∼ Leftward, Rightward[Table table17-23312165261422955]

**Table table17-23312165261422955:**

Factor	Sum Sq	Mean Sq	NumDF	DenDF	F Value	Pr(>F)
Group	21171.29	21171.29	1	45.51881	8.82535	0.004731
Condition	42182.91	21091.46	2	347.1191	8.792073	0.000188
Position change	86550.14	21637.53	4	340.9407	9.019707	6.19E-07
Direction	7046.682	7046.682	1	341.2303	2.937443	0.087456
Group:Position change	3837.56	959.3901	4	340.8847	0.399926	0.808678
Condition:Position change	5116.538	639.5672	8	340.8213	0.266607	0.976245
Group:Direction	12259.14	12259.14	1	341.0939	5.110281	0.024414
Condition:Direction	11853.36	5926.679	2	341.2526	2.470564	0.086048
Position change:Direction	57547.91	14386.98	4	340.9649	5.997279	0.000113
Group:Position change:Direction	18393.1	4598.274	4	340.8851	1.916812	0.10719
Condition:Position change:Direction	19502.63	2437.829	8	340.7885	1.01622	0.423308
Group:Condition	#N/A	#N/A	#N/A	#N/A	#N/A	#N/A
Group:Condition:Position change	#N/A	#N/A	#N/A	#N/A	#N/A	#N/A
Group:Condition:Direction	#N/A	#N/A	#N/A	#N/A	#N/A	#N/A
Group:Condition:Position change:Direction	#N/A	#N/A	#N/A	#N/A	#N/A	#N/A

Linear mixed-effects model: Prop. (L1) ∼ Group*Listening condition*Binned speaker position*Direction of movement + (1|IDs)

Proportion of time spent moving in same direction as speaker (%) during stationary sound localization (L1)

Group (2 levels) ∼ Control, BCD

Listening condition (4 levels) ∼ Binaural (Control), Left only (BCD), Right only (BCD), Bilateral (BCD)

Binned speaker position (12 levels) ∼ −60° to (−50°), …, 50° to 60°[Table table18-23312165261422955]

**Table table18-23312165261422955:**

Factor	Sum Sq	Mean Sq	NumDF	DenDF	F Value	Pr(>F)
Group	10013.85	10013.85	1	66.61002	9.837016	0.002546
Condition	1550.572	775.286	2	418.9631	0.761595	0.467566
Speaker position	19512.14	1773.831	11	410.5153	1.742506	0.062223
Group:Speaker position	22442.43	2040.221	11	410.7864	2.004192	0.026731
Condition:Speaker position	30377.9	1380.814	22	410.5823	1.35643	0.131105
Group:Condition	#N/A	#N/A	#N/A	#N/A	#N/A	#N/A
Group:Condition:Speaker position	#N/A	#N/A	#N/A	#N/A	#N/A	#N/A

Linear mixed-effects model: Prop. (L1-to-L2) ∼ Group*Listening condition*Direction of movement*Position change + (1|IDs)

Proportion of time spent moving in same direction as speaker (%) during moving sound localization (L1-to-L2)

Group (2 levels) ∼ Control, BCD

Listening condition (4 levels) ∼ Binaural (Control), Left only (BCD), Right only (BCD), Bilateral (BCD)

Position change (5 levels) ∼ 0°, ±20°, ±40°[Table table19-23312165261422955]

**Table table19-23312165261422955:**

Factor	Sum Sq	Mean Sq	NumDF	DenDF	F Value	Pr(>F)
Group	5039.975	5039.975	1	188	4.741313	0.030692
Condition	4250.535	2125.267	2	188	1.999327	0.138296
Position change	22562.94	5640.735	4	188	5.306473	0.000453
Group:Position change	6807.864	1701.966	4	188	1.60111	0.175749
Condition:Position change	3831.969	478.9961	8	188	0.450611	0.88908
Group:Condition	#N/A	#N/A	#N/A	#N/A	#N/A	#N/A
Group:Condition:Position change	#N/A	#N/A	#N/A	#N/A	#N/A	#N/A
